# A Comprehensive Review on Finite Element Analysis of Laser Shock Peening

**DOI:** 10.3390/ma17174174

**Published:** 2024-08-23

**Authors:** Mayur B. Wakchaure, Manoranjan Misra, Pradeep L. Menezes

**Affiliations:** 1 Department of Mechanical Engineering, University of Nevada, Reno, NV 89557, USA; mwakchaure@unr.edu; 2 Department of Chemical and Materials Engineering, University of Nevada, Reno, NV 89557, USA; misra@unr.edu

**Keywords:** laser shock peening, finite element analysis, residual stresses

## Abstract

Laser shock peening (LSP) is a formidable cold working surface treatment that provides high-energy precision to enhance the mechanical properties of materials. This paper delves into the intricacies of the LSP process, offering insights into its methodology and the simulation thereof through the finite element method. This review critically examines various points, such as laser energy, overlapping of shots, effect of LSP on residual stress, effect of LSP on grain refinement, and algorithms for simulation extrapolated from finite element analyses conducted by researchers, shedding light on the nuanced considerations integral to this technique. As the significance of LSP continues to grow, the collective findings underscore its potential as a transformative technology for fortifying materials against mechanical stress and improving their overall performance and longevity. The discourse encapsulates the evolving landscape of the LSP, emphasizing the pivotal role played by finite element analysis in advancing our understanding and application of this innovative surface treatment.

## 1. Introduction

Laser shock peening (LSP) is a surface enhancement technique used to improve the mechanical properties of materials, particularly metals, by inducing compressive residual stresses through the application of laser-induced shock waves. The research conducted by Askar’yan and Moroz in 1962 provides significant insights into the effect of high-intensity lasers on materials, particularly on a microscopic scale [1]. The essence of their findings lies in the profound impact of intense laser beams on tiny surface areas, leading to the phenomenon of material evaporation and the subsequent generation of recoil pressure [1]. In a study conducted in early 1967, researchers aimed to ascertain the criteria necessary for safely generating high beam power dissipation and to understand its various effects. The investigation delved into several key aspects, including heat transfer rates, nucleate and film boiling conditions, thermal stress, thermal shock, and fatigue [2]. In 1993, an extensive study was carried out to delve deeper into the impact of laser shock processing on low-carbon steel, specifically focusing on its microstructure and mechanical properties. The investigation sought to ascertain how this treatment influenced steel behavior and characteristics. The findings of this study revealed that while laser shock processing brought about notable modifications in certain aspects of the material, particularly its microstructure and mechanical properties, the observed benefits, particularly in terms of enhancing fatigue and wear resistance, did not reach the levels achieved by conventional shot-peening techniques [3]. The process described involves the utilization of a high-energy laser pulse directed onto a designated surface, which has been coated with a specific medium, such as water, black paint, or quartz mylar tape. This coating serves a crucial role in the process, acting as a confinement medium with which the laser energy can interact. When the high-energy laser pulse contacts the coated surface, it rapidly vaporizes the medium, generating an intense burst of energy. This energy generates a high-pressure wave that propagates through the material and induces a shock effect. This shock wave, propelled by the sudden release of energy, travels through the material, potentially leading to various outcomes depending on the intended application. This process underlines the precision and control achievable by harnessing high-energy laser technology, offering opportunities for diverse applications across industries ranging from manufacturing to scientific research and beyond [4]. In the initial phase of laser shock treatment, which marked the first generation, the utilization of iodine and neodymium glass lasers emerged as the premier choice for effectively treating metal joints, particularly within aircraft applications, owing to their exceptional performance and suitability for the task at hand [5]. Following its initial usage, then there afterward it has been incorporated into a plethora of applications, particularly in the biomedical field, where it is extensively employed for studies related to implant materials, showcasing its versatility and significance in advancing scientific research and technological developments aimed at improving healthcare outcomes [6,7]. LSP has advantages over chemical surface treatments like coatings, carburizing, and nitriding, particularly in terms of environmental sensitivity and durability.

Unlike chemical treatments such as coatings, carburizing, and nitriding, which are vulnerable to harsh environments, LSP introduces deep compressive residual stresses without altering the surface chemistry, making it more stable and reliable under varying conditions [7]. LSP does not introduce surface contaminants or require high temperatures, thereby minimizing the risk of thermal distortion or oxidation and enhancing the performance of the treated components. By employing high-energy laser pulses, LSP effectively induces compressive residual stresses and improves surface properties, such as fatigue strength and hardness. Unlike carburizing and nitriding, which focus on altering surface chemistry to increase hardness, LSP’s effectiveness in enhancing fatigue life is particularly notable in challenging environments where chemical treatments might be less effective. Thus, LSP offers a versatile and robust alternative for industries that demand high performance under severe conditions.

LSP distinguishes itself from other peening techniques like Shot Peening (SP) and Ultrasonic Impact Peening (UIP) through its ability to induce deep compressive residual stresses, often extending several millimeters below the surface. This capability significantly enhances the fatigue life and resistance to stress corrosion cracking (SCC). Unlike SP, which can induce deep stresses but may also roughen the surface and negatively affect fatigue and corrosion resistance, LSP typically maintains or even improves the surface finish. This makes LSP particularly suitable for high-precision components in critical industries, such as aerospace and nuclear. While UIP is effective at improving the surface finish and reducing porosity, especially in additively manufactured parts, it generally produces shallower compressive stresses compared to LSP. Overall, LSP’s ability to deliver deep impact with minimal surface alteration provides a distinct advantage for applications demanding high durability and precision [7]. Since its inception in the 1960s, laser technology has made significant strides, particularly in the aerospace sector [5].

The primary goal behind the utilization of LSP lies in its multifaceted advantages aimed at enhancing the structural integrity and performance of metals and alloys. LSP serves as a pivotal technique to augment fatigue life, which is a critical aspect in ensuring prolonged durability under cyclic loading conditions. Moreover, its application facilitates a notable enhancement in corrosion resistance, which is a vital attribute of materials subjected to harsh environmental conditions, thereby extending their service life. Additionally, LSP imparts a significant increase in the yield strength, thereby fortifying the material against mechanical stress and potential failure. Furthermore, it contributes to the refinement of the surface roughness, leading to improved mechanical properties and surface integrity. Lastly, the process of LSP plays a pivotal role in refining the microstructure of metals and alloys, enhancing their overall mechanical properties and performance characteristics, making it an indispensable technique in various industrial applications demanding superior material performance and longevity [8,9,10].

LSP is a proven technique used to mitigate crack propagation in various materials. By subjecting the surface of a specimen to high-intensity laser-induced shock waves, LSP induces beneficial compressive residual stresses that oppose crack initiation and growth. These compressive residual stresses act to stabilize the material, effectively reducing or delaying the propagation of cracks. This method is particularly effective for enhancing the fatigue life and durability of critical components in aerospace, automotive, and other high-performance engineering applications. Therefore, the application of LSP serves as an invaluable tool for improving the structural integrity and reliability of materials subjected to mechanical loading [11].

The LSP process is commonly employed for the surface treatment of materials; as a result, plastic deformation occurs on the specimen’s surface. This deformation manifests as a restructuring of the material at the atomic level, leading to the introduction of strains within the material. These strains play a pivotal role in determining the various mechanical properties and performance characteristics of the material. Plastic deformation induced by LSP can result in changes in surface hardness, residual stress distribution, and fatigue resistance, making it a crucial factor in assessing the effectiveness of the LSP treatment. Understanding and controlling the extent of plastic deformation is essential for optimizing the desired outcomes of LSP, particularly in applications where surface integrity and durability are paramount. Therefore, a detailed analysis and characterization of the plastic deformation induced by LSP are critical for informing material design and engineering decisions in various industrial contexts.

The influence of external temperature on LSP outcomes has been the subject of investigation, with findings indicating a notable impact on the effectiveness of the process. Research has revealed that conducting LSP procedures under elevated temperature conditions can compromise the desired strengthening effects. This phenomenon highlights the importance of considering temperature variables in the application of LSP techniques, as they can significantly alter the material response and overall efficacy of the treatment. Understanding and accounting for these thermal effects are crucial for optimizing the outcomes of LSP applications across various industrial contexts, where precise control over material properties is paramount. Therefore, practitioners and researchers alike must acknowledge and address temperature considerations to ensure the reliability and success of LSP interventions [12].

Research on scanning patterns and their impact on residual stresses is limited, and only a few studies have addressed this topic. One notable study conducted by Samuel et al. [12] explored the effects of scanning patterns on residual stresses. Their findings suggested that employing the L-spiral scanning technique resulted in significant improvements in fatigue life. This indicates the potential of L-spiral scanning to positively influence the structural integrity and performance of components subjected to cyclic loading. Further investigation of scanning strategies and their influence on residual stresses could offer valuable insights into optimizing additive manufacturing processes and enhancing the mechanical properties of printed parts [13]. Scanning patterns play a significant role in profiling. However, it is noted that while scanning patterns contribute substantially to the profile, their limitations and non-uniformity do not have a profound impact. This suggests that while scanning patterns influence the formation of a profile, factors such as constraints and irregularities within these patterns do not significantly alter the overall profile outcome. This insight underlines the importance of considering scanning patterns in profiling methodologies while acknowledging their inherent limitations and variations. Such an understanding can inform the development of more robust profiling techniques that account for these nuances effectively [14].

The understanding of LSP phenomena and their effects on material properties is crucial for optimizing the process parameters and predicting the resulting material response accurately. In this context, the finite element method (FEM) serves as a powerful computational tool for analyzing LSP processes. By discretizing the material into finite elements and solving the governing equations, FEM enables the simulation of complex physical phenomena, such as shock wave propagation and its interaction with the material. Previous research endeavors have employed FEM to investigate LSP, particularly focusing on predicting the magnitude of residual stresses induced in specific materials, such as Ti-6Al-4V, a commonly used titanium alloy known for its high strength-to-weight ratio and corrosion resistance. These studies contribute valuable insights into the underlying mechanisms of LSP and provide essential data for optimizing the process parameters to effectively enhance material performance [15]. The paper begins with an LSP introduction and then, in consecutive sections, delves into the key parameters that govern the effectiveness of the LSP process, focusing on critical aspects such as laser parameters, overlapping of laser shots, and the significance of spot size in achieving optimal results. Following this, this paper examines the effects of LSP on the material, particularly its role in improving the mechanical properties and promoting grain refinement. The Discussion then transitions into the use of finite element analysis (FEA) algorithms to simulate and predict the outcomes of the LSP process, highlighting the influence of meshing on simulation accuracy. This paper further explores advancements in LSP FEA, offering a progressive perspective on how these innovations are shaping the field. The Discussion section synthesizes the findings and implications of the research, setting the stage for future directions where potential areas for further exploration and development are identified. The paper concludes with a summary of the key points discussed, reinforcing the significance of LSP and its simulation in advancing material science and engineering. Building on this understanding, it is necessary to explore the practical aspects of the LSP process itself. The following section outlines the key components and steps involved in the LSP process.

## 2. LSP Process

Initially, the setup involves the integration of key components: a device responsible for generating the laser beam, a confinement film or layer, and the target material. During the LSP process, a series of essential steps unfold to achieve the desired outcomes. As outlined in the introductory section, the confinement layer plays a crucial role in this process. It acts as a barrier to contain the energy imparted by the laser, thereby ensuring effective transmission onto the target material. This confinement layer can vary in composition, with options including black paint, water, or aluminum foil, all of which are applied to the surface of the specimen. It is important to note that the selection of the confinement element significantly influences the shock wave dynamics generated on the specimen’s surface. Therefore, careful consideration and optimization of this aspect are paramount for the overall success and efficacy of the LSP process [16]. The shock wave phenomenon is characterized by an extremely brief duration of 10 to 20 nanoseconds (ns). Shock waves are rapid changes in pressure and density that propagate through a medium and typically arise from events such as explosions, supersonic projectiles, or intense energy release [17]. Experimental studies have revealed a significant contrast in the expansion behavior of plasma between two distinct media, namely air and water. Specifically, the expansion of plasma in air surpasses that in water by a factor of 20. This substantial variance in expansion dynamics has profound implications, particularly in the context of shockwave propagation. The heightened expansion observed in air results in a more extensive spread of the shock wave, which in turn mitigates the pressure exerted on the surface upon which the plasma interacts. This observation emphasizes the critical role of the surrounding medium in influencing the behavior and effects of plasma expansion, with implications for various technical applications and phenomena [18]. The process described aims to induce a residual compressive stress field within metallic specimens by utilizing the propagation of laser-induced high-pressure plasma. This technique involves several sequential steps to achieve its objective. Initially, a laser is employed to generate high-pressure plasma, which serves as the medium for transmitting the compressive stress onto the metal surface. As the plasma expands, it exerts pressure on the material, creating a compressive stress field. This stress field is crucial for enhancing the mechanical properties and fatigue resistance of metallic components, making them more durable and reliable in various applications. By carefully controlling the parameters of the laser and plasma generation, precise and consistent results can be achieved, ensuring the effectiveness of the process. Overall, this method offers a promising approach for imparting desirable residual stresses to metallic specimens, contributing to the improvement of their performance and longevity in engineering and manufacturing contexts. Initially, when laser pulses with intensities reaching gigawatts per square centimeter (GW cm^−2^) strike the surface of a metal confined within a specific element, they induce the process of ablation, removing a very thin layer of the surface, typically less than 1 µm per shot. Subsequently, the vapor generated from this ablation process continues to interact with the incoming laser energy, leading to ionization and the formation of high-pressure plasma. This plasma experiences amplification in pressure, reaching levels of several gigapascals (GPa) owing to the confining effect exerted by the surrounding water. Because of this amplified pressure, a significant pressure discontinuity arises, which propagates into the material as a shockwave. This shock wave phenomenon plays a pivotal role in various applications, especially in material processing and manipulation, as it induces profound changes within the material’s structure and properties, making it a crucial aspect in the realm of laser-matter interaction studies and technological advancements [19,20,21].

**Figure 1:**
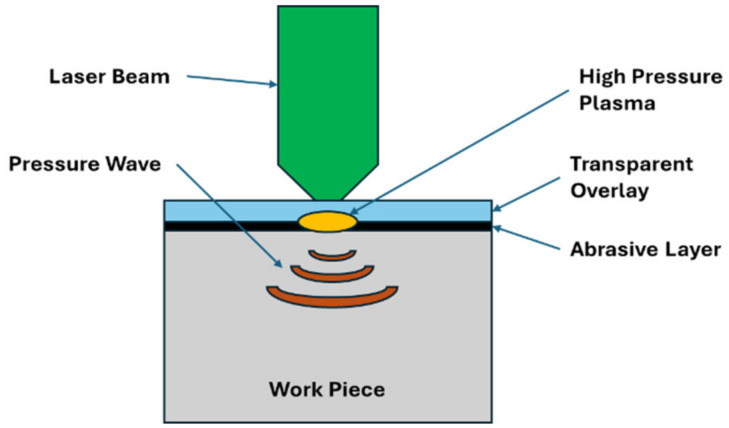
Schematic of the LSP process.

The outcome of a process wherein a metallic specimen undergoes heterogeneous plastic deformation leads to the development of induced compressive stresses within the material. This deformation involves the alteration of the specimen’s shape due to the application of external forces, resulting in a non-uniform distribution of stress and strain across its structure. The term “heterogeneous” implies that this deformation is not uniform throughout the material but varies spatially, possibly due to variations in the material’s composition, microstructure, or the application of complex loading conditions. The development of compressive stresses signifies that the deformation tends to decrease the specimen’s volume, as opposed to tensile stresses, which would elongate it. Understanding such phenomena is crucial in various engineering applications, including material testing, structural design, and manufacturing processes, where knowledge of how materials respond to mechanical forces is essential for ensuring the integrity and performance of components [16]. The plasma-confined regime, characterized by its ability to confine and manipulate plasma, demonstrates a significant exertion of maximum impact pressure reaching up to 5 gigapascals (GPa) within the intensity range of 8 to 10 gigawatts per square centimeter (GW cm^−2^). The effectiveness was empirically validated through experimental investigation, highlighting its capability to generate intense pressure within a short pulse duration of 10–20 nanoseconds (ns). Such high-pressure conditions are pivotal for various technical applications, particularly in fields such as materials science, fusion research, and high-energy physics. This experimental confirmation underscores the practical relevance and potential of the plasma-confined regime for harnessing controlled pressure dynamics for diverse technological advancements [21]. Figure 1 shows a schematic of the LSP process.

In the realm of investigating LSP through finite element analysis (FEA), one of the pioneering endeavors involved the anticipation of residual stress distribution and the subsequent optimization of stress parameters. This seminal study, dating back to 1999, was spearheaded by Braisted and Brockman [22], employing Abacus/Explicit software Version 5.7; they delved into the intricacies of LSP on carbon steel. Through thorough simulation and analysis, they aimed to comprehend the nuanced interplay of laser-induced shockwaves with the material properties of carbon steel, particularly focusing on the resultant stress fields. Their utilization of advanced computational tools signified a crucial leap forward in understanding the mechanics and potential applications of LSP, paving the way for subsequent research and development in this domain. In the realm of plasma physics, the phenomenon of plasma explosion unfolds within an incredibly brief timeframe, typically spanning nanoseconds. During this fleeting period, the material involved undergoes significant deformation, manifesting in a plastic manner. Consequently, to accurately model and analyze such nonlinear behavior, researchers resort to employing explicit methods within their computational frameworks. This choice is particularly suitable for addressing the complexities inherent in the field of LSP. LSP is characterized by its short-lived dynamic nature, marked by the rapid propagation of elastic-plastic stress waves. These waves encompass a spectrum of events ranging from swift impacts to the subsequent propagation of waves through the material. Given the rapidity and intricacies of these phenomena, conventional numerical techniques often prove inadequate. Hence, explicit time integration FEA software’s or codes have emerged as indispensable tools for effectively capturing the intricacies of LSP dynamics. By leveraging these specialized computational methodologies, researchers can delve deeper into understanding the mechanics governing plasma explosions and similar dynamic processes, thereby facilitating advancements across various scientific and engineering domains [23]. Having established the importance of using explicit methods for modeling LSP dynamics, it is crucial to now turn our attention to the key parameters that influence the LSP process.

## 3. Key Parameters for the LSP Process

### 3.1. Laser Parameters

In LSP, the magnitude of the laser energy plays a pivotal role, serving as a critical determinant in the generation of shock waves. Researchers have established a direct correlation between the laser energy input and the resulting shock wave intensity. Specifically, the shock wave’s magnitude is predominantly contingent upon the amount of laser energy applied. Notably, investigations into this phenomenon have revealed that when water is employed as a confinement medium, the expansion of plasma exhibits a relationship with the square root of the laser intensity. This relationship is quantified in terms of gigawatts per square centimeter (GW cm^−2^), which elucidates the intricate interplay between laser energy and shock wave generation. Such insights into the fundamental dynamics of LSP are crucial for optimizing the process parameters and enhancing its efficacy in various technical applications [19,24]. Laser intensity, a crucial parameter in laser surface processing, plays a significant role in altering material properties. Its calculation involves dividing the laser energy by the area and then multiplying the result by the pulse width. Essentially, this formula highlights that the laser intensity can be augmented through two primary methods: boosting the laser energy or minimizing the focus area. Experimental observations highlight the profound impact of a heightened laser power density. Such augmentation causes a notable increase in grain refinement within materials, particularly in the case of LSP’s TC4 titanium alloy. This refinement phenomenon manifests through the formation of dislocation cells and subgrain boundaries, which denote structural alterations induced by laser treatment. These findings emphasize the intricate relationship between laser parameters and material responses, offering valuable insights for optimizing laser surface processing techniques and enhancing material properties for diverse applications [25]. When considering the effect of increasing the pulse energy on material behavior, it is essential to note its impact on both the penetration depth and residual stress. As the pulse energy escalates, it directly correlates with the heightened penetration depth into the processed material. This phenomenon occurs due to the increased intensity and duration of the energy delivered to the material surface, facilitating deeper penetration. Simultaneously, the higher pulse energy levels also contribute to generating a slightly elevated maximum compressive residual stress within the material structure post-processing. This increase in compressive residual stress can be attributed to the greater energy imparted onto the material, leading to more extensive deformation and redistribution of the material particles, ultimately resulting in higher compressive stresses. Understanding these effects of pulse energy variations is crucial for optimizing material processing parameters and achieving the desired material properties in various technical applications, particularly in fields such as laser processing, welding, and materials science [26].

**Figure 2:**
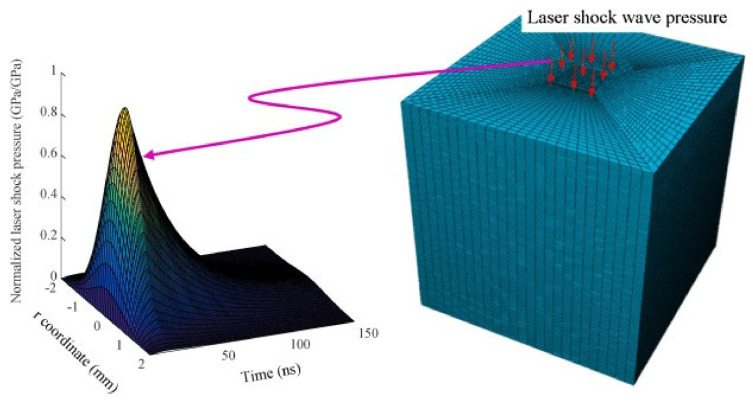
Laser shock wave pressure and finite element model of LSP [27] Copyright Elsevier, 2021.

The study conducted by Fabbro et al. [19] investigated the correlation between the pressure generated after a plasma explosion and the laser intensity employed. The variables under consideration include Io, which represents the power density of the laser, and P, which denotes the peak pressure resulting from the plasma explosion. Additionally, this study delves into the concept of Z, which represents reduced acoustic impedance, a critical parameter in understanding the acoustic behavior of the system. The equation presented, 2/Z = 1/Z1 + 1/Z2, elucidates the relationship between the acoustic impedances of the two different media involved in the process. This equation provides insight into the complex interplay between laser intensity, plasma explosion dynamics, and resultant pressure effects, contributing valuable information to the field of laser-plasma interactions and associated phenomena. Through their research, Fabbro et al. offered a nuanced understanding of how varying laser intensities influence the pressure dynamics post-plasma explosion, shedding light on fundamental principles crucial for diverse technical applications, ranging from material processing to advanced propulsion systems. The pressure generated by the incident laser is given in Equation (1) [19]. Figure 2 shows that the shock wave pressure profile in FEA model.

(1) $$P(GPa)=0.01\sqrt{\frac{\alpha }{2\alpha +3}}\sqrt{z({g\;cm}^{-2}\;s^{-1})}\sqrt{I_{0}(GW\;{cm}^{-2}})$$

By quantifying the critical pressure necessary for plastic deformation, engineers and researchers can better predict the behavior of materials under different loading conditions and optimize their performance in various applications, ranging from the aerospace to automotive industries. The term “HEL” refers to the specific designation for the minimum pressure required to induce plastic deformation [22]. It is based on the dynamic yield strength of base material [28] and the equation for HEL described by

(2) $$HEL=\frac{1-\upsilon }{1-2\upsilon }DYS$$

where HEL: Hugonoit Elastic Limit, υ: Poisson’s ratio, DYS: Dynamic yield strength.

In technical terms, when discussing material behavior under pressure, it is crucial to consider the concept of the HEL. The HEL represents the maximum pressure a material can withstand without undergoing permanent deformation. In practical terms, if the peak pressure applied to a material is below the HEL, the material will only experience elastic deformation, meaning that it will return to its original shape once the pressure is released. However, to induce plastic deformation, where the material undergoes permanent changes in shape, the peak pressure must exceed the material’s HEL. This distinction is essential for understanding how materials respond to varying levels of pressure and is particularly relevant in fields such as materials science, engineering, and mechanics, where the behavior of materials under stress is of paramount importance for designing and analyzing structures and components. Therefore, careful consideration of the relationship between peak pressure and HEL is necessary to ensure the integrity and performance of materials subjected to high-pressure environments or mechanical stress [29]. In technical parlance, there is a consensus that an optimal pressure range for the LSP parameter typically falls within the spectrum of 2 to 2.5 times the HEL. This benchmark serves as a crucial determinant in the application of the LSP. The significance of adhering to this specific pressure range lies in its direct correlation with the effectiveness and efficiency of the peening process. By maintaining the pressure within this prescribed range, researchers can ensure the desired outcome of introducing beneficial compressive stresses to the material surface, thereby enhancing its fatigue life, corrosion resistance, and overall mechanical integrity. Moreover, the pressure created directly depends on the laser input energy, as seen in Equation (1). This consensus on the optimal pressure range underscores the calibration and precision required in the implementation of LSP techniques, emphasizing the importance of adhering to established parameters to achieve the desired material performance improvements [30]. In FEA, the pressure computed through the Johnson–Cook (JC) equation serves as a crucial input parameter for simulating the behavior of materials under various conditions. The JC equation is particularly valuable for predicting the material response under high-strain-rate conditions, such as those encountered in dynamic loading scenarios or machining processes. By applying the calculated pressure derived from the JC equation as an input in FEA simulations, engineers can obtain insightful results regarding the structural integrity, deformation, and stress distribution within the analyzed components or structures.

To comprehensively investigate plastic deformation through FEA, a thorough understanding of the dislocation equation employed in programming is imperative. Dislocations, which are crucial defects in crystalline structures, significantly influence the mechanical behavior of materials, especially in plastic deformation scenarios. By incorporating the dislocation equation into FEA programming, analysts can accurately predict the material behavior, including the initiation and propagation of dislocations within the crystal lattice. Understanding the intricacies of this equation facilitates the simulation of dislocation dynamics, aiding the elucidation of deformation mechanisms and the prediction of material strength, ductility, and other mechanical properties. Hence, a robust comprehension of the dislocation equation within the context of FEA programming not only enhances the fidelity of simulations but also contributes to advancements in materials science and engineering, enabling the development of innovative designs and improved performance of structural components [27].

Below are the details of the laser parameters that influence the efficiency of the LSP process, leading to the modification of the material properties.

I. Laser Intensity
  A higher laser intensity generates stronger shock waves, leading to deeper compressive residual stresses and improved fatigue resistance. This is because the increased energy density enhances the plastic deformation of the material, resulting in a more significant alteration in its microstructure. However, excessive intensity can cause surface damage or melting, which can compromise the integrity of the treated material and lead to undesirable effects such as micro-cracking or surface roughness. A lower laser intensity produces milder shock waves, which may not penetrate as deeply, resulting in less pronounced improvements in material properties. Although this can be beneficial for treating delicate or thin materials, where excessive energy might cause damage, it may not provide the same level of enhancement in fatigue resistance or hardness as higher intensities [31,32,33].
II. Pulse Duration
  A shorter pulse duration typically results in higher peak pressures, enhancing the depth and magnitude of the compressive residual stresses. This is because shorter pulses concentrate the energy in a brief time frame, creating intense shock waves that penetrate deeper into the material. These high peak pressures can significantly improve the material’s fatigue resistance and hardness. However, very short pulses can lead to surface ablation, where the material is removed from the surface due to the high-energy density, potentially causing surface roughness or damage. A longer pulse duration generates lower peak pressures, which might reduce the effectiveness of the peening process in terms of the depth and magnitude of the compressive residual stresses. The energy is spread over a longer period, resulting in less intense shock waves. However, this can be beneficial for treating more delicate materials, as it reduces the risk of surface damage and ablation. Longer pulses can also provide a more uniform treatment over larger areas, which is advantageous for applications requiring gentle processing [34,35,36].
III. Spot Size
  A smaller laser spot size concentrates the energy over a limited area, resulting in a higher peak stress. This intense focus can be particularly useful for localized strengthening because it enhances material properties in specific regions. A higher energy density in a smaller spot size can lead to more significant plastic deformation and deeper compressive residual stresses. However, limited coverage means that only a small area benefits from these high stresses, which might require multiple passes to treat larger surfaces effectively. Conversely, a larger laser spot size distributes energy over a broader area, leading to a more uniform stress distribution. This can be advantageous for treating larger surfaces more evenly, reducing the likelihood of untreated areas, and ensuring consistent material properties across the entire surface. However, the peak stresses are generally lower compared to those achieved with a smaller spot size, which might result in less pronounced improvements in localized material properties. The trade-off between spot size and stress distribution is crucial for optimizing the laser treatment process based on specific applications and material requirements [37,38].
IV. Coverage
  A higher laser coverage ensures a more uniform treatment and consistent material properties across the surface. This uniformity is crucial for enhancing the mechanical properties of the material, such as hardness and wear resistance. It can improve the overall fatigue resistance by creating a more homogeneous microstructure, which helps distribute stress more evenly and reduces the likelihood of crack initiation. Additionally, higher coverage minimizes the risk of untreated areas, which can act as stress concentrators and lead to premature failure. On the other hand, lower laser coverage may leave some areas untreated, leading to an uneven stress distribution and potentially weaker spots. These untreated areas can become focal points for stress, increasing the risk of fatigue and failure under cyclic loading. Inconsistent treatment can also result in variations in the surface hardness and wear resistance, compromising the overall performance of the material [39,40,41].
V. Wavelength
  In laser shock peening, shorter wavelengths are generally absorbed more efficiently by the material, leading to more effective shockwave generation. This increased absorption can enhance the peening effect, improving the surface hardness and residual stress distribution. However, the higher energy concentration associated with shorter wavelengths can also elevate the risk of surface damage, such as melting or ablation. Conversely, longer wavelengths penetrate deeper into the material, which can be beneficial for treating subsurface layers and achieving a more uniform stress distribution throughout the material. However, these longer wavelengths often require higher laser intensities to generate shock waves of strengths comparable to those produced by shorter wavelengths. This can make the process less efficient and potentially more costly due to the need for more powerful laser systems. Additionally, the choice of wavelength can influence the overall effectiveness of the peening process, depending on the specific material properties and desired outcomes. For instance, materials with higher absorption coefficients for shorter wavelengths may benefit more from their use, while those requiring deeper penetration might be better suited for longer wavelengths [32,42].

### 3.2. Overlapping of Laser Shots

**Figure 3:**
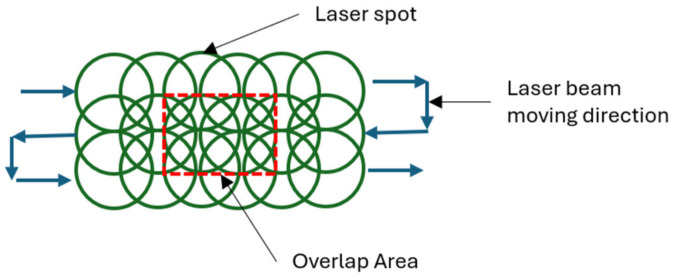
Overlapping of laser beam.

A recent study focused on optimizing the overlapping ratio of laser shows revealed significant findings pertaining to stress dynamics. The research demonstrates that as the overlap between laser beams increases, there is a corresponding escalation in stress levels. Particularly noteworthy is the observation of heightened tensile stresses exerted on the surface with an increase in the overlap ratio. This suggests a direct correlation between the extent of overlap and the magnitude of stress experienced. Such insights are crucial for various technical applications in which laser systems are utilized, offering valuable guidance for enhancing performance and mitigating potential structural concerns. Understanding the intricate relationship between overlapping ratios and stress distribution is pivotal for ensuring the reliability and longevity of systems reliant on laser technology. These findings highlight the importance of careful optimization processes in engineering design and the nuanced interplay between operational parameters and material behavior in laser-based applications [43]. A schematic diagram showing the overlapping of shots is shown in Figure 3.

The overlapping rate, within the context of residual compressive stress fields, is a pivotal parameter exerting a significant influence on the consistency and thickness of the said stress field. This parameter plays a critical role in determining the effectiveness and reliability of processes involving residual compressive stress, particularly in applications such as material strengthening or surface modification. The degree to which overlapping occurs directly impacts the uniformity and intensity of the compressive stress distributed across a material or surface. A higher overlapping rate typically results in a more consistent and thicker residual compressive stress field, enhancing desired properties such as fatigue resistance, wear resistance, and overall structural integrity. Therefore, careful control and optimization of the overlapping rate become imperative in engineering practices where the manipulation of residual compressive stress fields is integral to achieving the desired performance outcomes. Understanding and appropriately managing this parameter significantly contributes to the success and efficacy of various technological advancements and industrial applications reliant on residual stress engineering [44,45].

As the overlapping rate between components or materials increases, it results in a proportional increase in the compressive stress layer. This phenomenon is particularly significant in structural and material engineering contexts, where overlapping interfaces play a crucial role in distributing forces and maintaining structural integrity. The increase in the overlapping rate leads to a more pronounced compression of the material or component at the interface, effectively enhancing its ability to withstand external loads or forces. Understanding this relationship is essential for optimizing designs in various fields, such as composite materials, adhesive bonding, and mechanical joints. Engineers and researchers often utilize this knowledge to improve the performance and durability of structures subjected to mechanical stress, thereby enhancing their safety and reliability in diverse applications, ranging from aerospace engineering to civil infrastructure. Furthermore, precise quantification and analysis of the relationship between the overlapping rate and compressive stress layer are imperative for predictive modeling and simulation efforts aimed at optimizing designs for specific performance criteria and operational conditions [46]. This statement suggests that an increase in overlapping, presumably referring to overlapping elements or processes, results in the enhancement of surface stress homogeneity. This enhancement occurs through the mechanism of smoothing the surface stress. In technical terms, surface stress homogeneity implies a uniform distribution or consistency of stress across the surface under consideration. When overlapping increases, this implies a greater convergence or interaction of elements, which in turn tends to equalize or distribute the stress more evenly across the surface. The process of smoothening the surface stress likely involves the reduction of irregularities or fluctuations in stress levels, resulting in a more uniform stress profile. This phenomenon could have implications in various technical areas where surface stress plays a significant role, such as material science, engineering, or mechanics. Understanding how increasing overlapping affects surface stress homogeneity can inform the design and optimization of structures, materials, or processes to achieve the desired performance or characteristics. Further research and experimentation may be necessary to fully comprehend the underlying mechanisms and potential applications of this phenomenon in specific technical domains [47].

According to the findings derived from the computational analysis conducted utilizing Ansys/LS-Dyna on a nickel-based single-crystal superalloy, it has been determined that implementing a 50% overlap rate can effectively result in a uniform distribution of surface cold rolled stripes (CRS). This observation has significant implications for the structural integrity and performance of components made from such alloys, as the uniform distribution of CRS ensures consistent material properties across the surface. The utilization of advanced computational tools like Ansys/LS-Dyna enables engineers and researchers to accurately model and simulate complex material behaviors under various conditions, facilitating the optimization of manufacturing processes and the enhancement of component reliability. By understanding the impact of overlap rates on the surface CRS distribution, practitioners can make informed decisions regarding alloy design and processing to meet performance requirements in industries such as aerospace, automotive, and power generation. This insight emphasizes the importance of computational analysis in advancing materials science and engineering, providing valuable guidance for the development of high-performance alloys tailored for demanding applications [48].

In certain instances, the physical alignment of a laser shot can result in a complete overlap, reaching a maximum of 100%. This phenomenon has significant implications, particularly in terms of the peak compressive stress generated and its impact on the plastic deformation depth. When such overlap occurs, the peak compressive stress experienced by the material being worked on increases. Consequently, this heightened stress level influences the extent to which plastic deformation occurs within the material, leading to deeper alterations in its structure and properties. This observation underlines the importance of precise laser control and shot placement to achieve the desired outcomes while minimizing unintended effects on material integrity and performance [49,50].

Table 1 summarizes the key parameters and their effects on the material properties.

Building on these insights into the influence of laser parameters, it is essential to explore the effect of LSP on material properties, particularly the role of residual stresses in depth.

## 4. Effect of LSP on Material Properties

Residual stresses derived from the outcomes of laser shock processing programs offer a significant avenue for enhancing the fatigue resistance of metals, particularly in aerospace applications. The efficacy of these parameters has been demonstrated in improving the fatigue properties of fastener regions within aircraft, especially when considering production conditions. By strategically applying laser shock processing techniques, specific system properties conducive to bolstering fatigue resistance can be achieved. This approach underlines the importance of residual stresses in fortifying metal structures against fatigue failure, thus contributing to the overall durability and reliability of aerospace components. Consequently, leveraging residual stresses in this manner presents a promising avenue for enhancing the performance and longevity of critical aircraft components, thereby advancing the safety and efficiency of aerospace systems [5].

A study has revealed the presence of residual tensile stress in a distinct zone near the center of the shot’s surface. This finding emphasizes the intricate dynamics during the shot process, indicating that even after the immediate impact, residual effects persist. Moreover, this research highlights the occurrence of stress oscillation, which is particularly noticeable at the shot’s periphery. Such oscillations denote a dynamic interplay of forces within the shot, potentially influencing its structural integrity and performance. Understanding these phenomena is crucial for optimizing shot design and manufacturing processes as well as ensuring the reliability and longevity of shot-based applications across various industrial situations. This insight into the distribution and behavior of stress within a shot contributes to advancing our knowledge of material behavior under dynamic loading conditions, thereby facilitating the development of robust and efficient shot technologies [28].

In technical frameworks, crack initiation mainly occurs due to elevated local tensile stresses induced by dynamic loading conditions. Such circumstances often lead to the propagation of cracks within materials, posing significant challenges to their structural integrity and durability. To mitigate this issue, various peening techniques have been devised and implemented in engineering practice. These techniques are strategically employed to induce compressive stresses within a material, thereby counteracting the detrimental effects of tensile stress. By subjecting the material’s surface to controlled mechanical impacts or shot blasting, peening processes effectively introduce compressive residual stresses, which help to impede crack initiation and propagation. This proactive approach serves as a preventive measure against structural failure and enhances the fatigue life of components subjected to dynamic loading. Consequently, the utilization of peening techniques is a crucial aspect of material engineering and structural design, ensuring the resilience and longevity of critical components in diverse industrial applications [51]. In an investigation conducted, a noteworthy observation was made regarding the formation of a tensile stress layer beneath the compressive region. This phenomenon denotes a structural response wherein a layer experiencing tensile stress is delineated beneath another layer that is subjected to compressive stress. Such a configuration often arises in structural systems or materials under varying loading conditions. The presence of this distinct tensile stress layer below the compressive region suggests a complex interplay between the forces and material behaviors within the system. Understanding this phenomenon is crucial for the analysis and design of structures to ensure their integrity and performance under diverse loading scenarios. Further research and analysis are warranted to elucidate the mechanisms driving the formation and characteristics of this tensile stress layer, offering valuable insights for engineering applications and advancements in material science [52].

**Figure 4:**
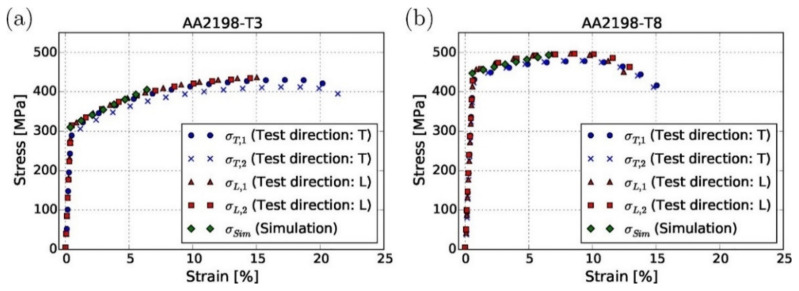
Stress–strain curves of AA2198-T3 (**a**) and AA2198-T8 (**b**) using flat tensile tests. The measured plastic material behavior is approximated using the Johnson–Cook model for the FE simulation shown as σ_Sim_ [52].

Tensile stress, a common concern in various applications, presents challenges due to its propensity to promote crack propagation within materials. However, employing LSP offers a promising solution that effectively mitigates these detrimental tensile stresses. Through careful control of the LSP process parameters, such as laser intensity, pulse duration, spot size, and coverage, engineers can strategically induce compressive stress on the material’s surface. This compression counteracts the tensile stress, creating a more balanced stress distribution within the material. Consequently, the likelihood of crack initiation and propagation is significantly reduced, thereby enhancing the structural integrity and durability of the component. This controlled application of compressive stress not only serves to alleviate existing tensile stresses but also acts as a preventive measure, delaying the onset of crack formation and extending the service life of the material. By leveraging LSP technology in this manner, engineers can effectively address the challenge of tensile stress-induced cracking, ensuring the reliability and longevity of critical components across various industries [17]. The process of parameter selection is of significant importance in the realm of controlling tensile stress, particularly in applications such as LSP. Given its criticality, there is a pressing demand for enhanced prediction methodologies to accurately anticipate the impact of selected parameters on stress levels. FEA has emerged as a pivotal tool in this context, offering a comprehensive framework for simulating and analyzing complex structural behavior. Researchers have increasingly leveraged FEA to broaden the horizons of stress prediction in LSP scenarios, thereby enabling more informed decision-making regarding parameter selection. Moreover, FEA has proven to be instrumental in advancing the field of fatigue life prediction, providing insights into the endurance characteristics of materials subjected to LSP-induced stresses. This integration of advanced computational techniques not only enhances our understanding of the underlying mechanics but also facilitates the optimization of LSP processes for improved performance and durability across various engineering applications [22,53,54]. Increasing the fatigue life of a material corresponds to an enhancement in its ability to withstand repeated loading cycles without failure, thereby contributing to its durability and longevity under mechanical stress. This improvement in fatigue life is often correlated with an increase in surface hardness, which is a property crucial for resistance against wear, abrasion, and deformation. The relationship between the fatigue life and surface hardness underscores the significance of the material properties in determining its performance in dynamic applications. By augmenting the surface hardness, the material becomes more resilient to the detrimental effects of cyclic loading, thereby extending its operational lifespan. This interconnectedness highlights the importance of considering both fatigue life and surface hardness as key factors in materials engineering and design processes, particularly in industries in which components are subjected to repetitive mechanical stresses. Achieving a balance between these properties can lead to the development of materials with superior mechanical characteristics and enhanced durability, ultimately optimizing the performance and reliability in various engineering applications [55]. Keller et al. [52] performed actual tensile testing on two LSP’ed specimens, and results in the form of a graph shown in Figure 4 consist of stress vs strain were compared with simulation results. This comparison shows that the average error was less than 5%.

Table 2 provides a detailed comparison of the simulation and experimental work performed on aluminum alloys by Keller et al. [52] and Yan H. et al. [50].

A comprehensive investigation carried out using FEM analysis has elucidated a notable correlation: as the frequency of peening impacts escalates, there is a corresponding augmentation observed in both the depth and magnitude of the residual compressive stress. This empirical observation emphasizes a crucial aspect within the realm of the materials field, shedding light on the intricate dynamics at play during the peening process. The application of FEM as a tool for this study not only offers quantitative insights but also facilitates a deeper understanding of the underlying mechanics governing the phenomenon. By systematically varying the parameters and simulating the peening procedure, researchers can discern a discernible trend, thereby enhancing their ability to predict and optimize the outcomes of such mechanical treatments. This finding holds significant implications for various industrial sectors reliant on surface enhancement techniques, providing valuable guidance for optimizing peening protocols to achieve the desired material properties and performance characteristics [56]. The phenomenon described entails a progressive rise followed by stabilization of the compressive residual stress values after a series of impacts. This trajectory suggests a temporal evolution wherein the compressive residual stress initially experiences an incremental escalation before reaching a point of equilibrium or steadiness. Such behavior may be indicative of a dynamic process wherein the material undergoes successive impacts, leading to gradual buildup and eventual stabilization of compressive residual stress levels. This observation holds significance in various technical domains, particularly in materials science and engineering, where understanding the behavior of residual stresses is crucial for assessing the structural integrity and performance of components subjected to impact loading. Investigating the mechanisms underlying this phenomenon can provide valuable insights for optimizing material properties and designing resilient structures capable of withstanding repetitive impacts while maintaining favorable stress distributions [48].

Part features, including elements such as holes or notches, have significant implications in stress concentration and the initiation of cracks within structural components. These features act as focal points for stress accumulation, where geometric irregularities disrupt the uniformity of the stress distribution across the material. Holes, for instance, introduce localized stress concentrations at their edges, particularly in scenarios of mechanical loading. Similarly, notches create stress concentration points due to abrupt changes in geometry, leading to heightened susceptibility to crack initiation. Understanding the impact of these features is crucial in engineering design and analysis processes because they influence the structural integrity and durability of components under various loading conditions. Engineers must meticulously consider the placement, size, and configuration of such features to mitigate potential failure risks and ensure the optimal performance and longevity of the respective parts. Consequently, comprehensive assessments of the stress distribution and crack propagation mechanisms in the vicinity of these features are imperative for ensuring the reliability and safety of engineering structures and systems [57].

The ASTM standard E647-11 is a crucial reference in the realm of fatigue crack growth rate analysis. This standard, developed by the American Society for Testing and Materials (ASTM), provides detailed guidelines and methodologies for assessing the rate at which cracks propagate under cyclic loading conditions. Specifically, it offers standardized procedures for conducting fatigue crack growth rate tests, ensuring the consistency and reliability of experimental results across different testing environments and materials. By adhering to ASTM E647-11, engineers and researchers can effectively evaluate the susceptibility of materials to fatigue failure, aiding in the design and maintenance of durable and safe structures and components in various industries, such as aerospace, automotive, and manufacturing. This standard serves as a cornerstone in fatigue analysis, facilitating informed decision-making processes and enhancing the overall integrity and performance of engineering systems [58].

In the realm of materials science and engineering, the significance of thickness in relation to fatigue life following LSP warrants attention. Through empirical investigation, it has been substantiated that the thickness of a specimen plays a pivotal role in determining its fatigue life post-LSP treatment. Intriguingly, the findings underscore a direct correlation between thinner specimen thickness and improved fatigue life outcomes. This implies that specimens with reduced thicknesses exhibit enhanced resilience against fatigue failure, thereby suggesting a potential avenue for optimizing the performance and longevity of materials subjected to LSP. Consequently, these insights serve as valuable technical reference points for researchers and practitioners alike, guiding the refinement of methodologies and formulation of strategies aimed at bolstering the fatigue resistance of materials in various engineering applications [59]. When implementing this technique, it is essential to acknowledge a potential consequence: an increase in the reflection of shockwaves. This outcome can have far-reaching effects, notably in the realm of inducing residual stress within the material under consideration. These residual stresses, in turn, hold the potential to significantly impact the grain refinement processes. Thus, while pursuing this method, it is imperative to carefully weigh the benefits against the potential drawbacks, particularly concerning the organization of shock wave reflection and the subsequent influence on residual stress formation and grain refinement within the material structure. Such considerations are paramount for ensuring the overall efficacy and integrity of the technical processes involved [60].

In technical terms, when the peak pressure within a system or structure attains a level of 2.5 times the HEL, it induces a significant plastic deformation. This deformation leads to the generation of reverse tensile stress, which is particularly noticeable at the central point of the shock. This occurrence is commonly referred to as the phenomenon of “residual stress hole”. Residual stress denotes the internal stress that remains within a material or structure even after the external forces causing deformation have been removed. The manifestation of this residual stress in the form of a ‘hole’ signifies a localized area where the stress has been particularly intense or concentrated. Understanding and managing residual stress holes are crucial in engineering and materials science, especially in applications where structural integrity and performance under varying loads are paramount. Engineers often employ sophisticated techniques such as FEA and stress simulations to predict and mitigate the effects of residual stress holes in order to ensure the reliability and longevity of components or systems subjected to high pressures or mechanical loads [30]. In the actual process of LSP, temperature emerges as a critical factor influencing residual stresses within materials. When the base material is subjected to elevated temperatures, a phenomenon known as stress relaxation occurs, wherein the stresses within the material gradually diminish over time. This thermal relaxation phenomenon is intricately tied to the degree of plastic deformation experienced during LSP. Essentially, the extent to which the material undergoes plastic deformation during the LSP process directly influences the rate and magnitude of stress relaxation observed at elevated temperatures. Therefore, understanding and controlling the temperature conditions during LSP operations are crucial for effectively dealing with residual stresses and ensuring the desired material properties and performance outcomes [61]. In addition to its influence on residual stresses, LSP also plays a significant role in microstructural changes within materials, particularly in the context of grain refinement.

## 5. Effect of LSP on Grain Refinement

**Figure 5:**
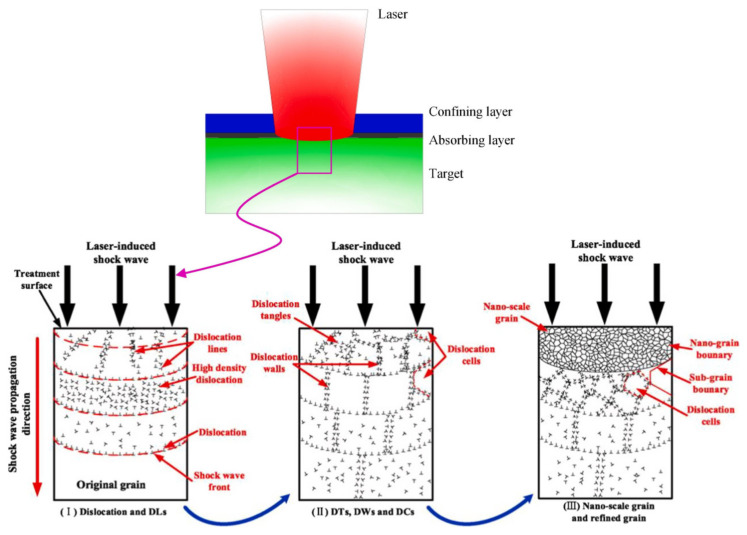
Schematic illustration of the surface nanocrystallization process of TC6 titanium alloy with high SFE induced by LSP [27] Copyright Elsevier, 2021.

In a comprehensive study conducted by Lou et al. [62] regarding the phenomenon of dislocation within microstructures during and after LSP, it was observed that the process induces dislocation movement along the surface of the specimen. This movement is primarily attributed to the propagation of waves generated during the peening process. Consequently, a significant increase in the density of dislocations occurs alongside the formation of dislocation-lined regions (referred to as DLs) within the original grain structure of the material. It is notable that the extent and nature of dislocation activity are heavily influenced by the stacking fault energy (SFE) characteristics of the material under consideration. This research highlights the intricate relationship between LSP-induced dislocation dynamics and material properties, providing valuable insights for understanding and optimizing the performance of materials subjected to such treatment processes. Several models have been developed to facilitate numerical calculations pertaining to grain refinement resulting from plastic deformation. These models serve as sophisticated computational tools. They are designed to simulate and analyze the intricate processes involved in grain refinement induced by plastic deformation, offering insights into the underlying mechanisms and behavior of materials under such conditions. By incorporating various parameters and equations derived from experimental data and theoretical principles, these models enable researchers and engineers to predict and understand the grain refinement phenomenon with a high degree of accuracy. Moreover, they provide a valuable platform for optimizing manufacturing processes, enhancing material properties, and advancing the development of novel materials with tailored microstructures. In technical literature and research, these models serve as essential references for studying the complex interplay between plastic deformation and grain refinement in diverse material systems, thereby significantly contributing to the advancement of material science and engineering knowledge [63,64,65]. In modern research, dislocation models have emerged as fundamental tools for forecasting grain refinement and dislocation behavior after the peening process. Peening induces plastic deformation in materials because of the impact of the high-velocity particles. The utilization of dislocation models in this situation has gained significant traction due to their efficacy in elucidating the intricate mechanisms underlying the evolution of the grain structure and dislocation distribution. By leveraging these models, researchers can better understand the microstructural changes induced by peening and tailor the process parameters to achieve the desired material properties. This approach not only enhances the predictive capabilities but also facilitates advancements in the development of high-performance engineering components with improved mechanical characteristics [66,67]. This phenomenon leads to the enhancement of surface properties, such as hardness and yield strength, in magnesium materials [67]. This phenomenon can be seen in the TC6 titanium alloy. Figure 5 elaborates more on the dislocation of wall grains and the effect of LSP on TC6 titanium alloy.

This improvement was attributed to the formation of a high-density dislocation array. Dislocations are line defects in the crystal lattice of materials, and their presence can significantly influence the mechanical properties. The high density of dislocations creates a more robust structure within the magnesium, which consequently leads to increased surface hardness and yield strength. This technical research emphasizes the importance of dislocation engineering in the manipulation and enhancement of material properties, particularly in magnesium alloys, thereby contributing to advancements in various engineering applications in which strength and durability are paramount [68]. To further understand and predict the material behavior under such conditions, computational tools like FEA techniques and algorithms are employed.

## 6. FEA Algorithms

The initial study involved the development and application of a Shockwave Finite Element (FE) code for analyzing the LSP process. Specifically, we focused on understanding the characteristics of the materials subjected to high strain rates. These material properties within the high-strain-rate region were utilized as key parameters within the rate-dependent plasticity algorithm. Through this approach, this study aimed to gain insights into the behavior of materials under dynamic loading conditions typical of LSP, which involves the generation of shockwaves through laser-induced impacts. This lysis framework not only facilitates a deeper understanding of the LSP process but also provides valuable technical data for optimizing and improving the efficiency of LSP applications in various industries [15]. In recent years, Python coding has become popular for solving engineering problems. One significant application of Python in engineering research was exemplified in a study conducted by Golabi et al. [51]. In their research, they applied the Particle Swarm Optimization (PSO) technique, leveraging Python coding within the Abaqus FE environment. The primary objective of their study was to enhance the uniformity of compressive residual stresses (RS) while concurrently minimizing the associated LSP cost, specifically focusing on an Inconel 718 super-alloy specimen. This integration of Python with Abaqus demonstrates its efficacy in optimizing complex engineering processes, offering a versatile and efficient tool for researchers and practitioners alike.

In technical analysis, the speed of the LSP process is typically measured in nanoseconds, achieving incredibly rapid strain rates of up to 10^6^ s^−1^. Such high-speed operations induce significant strain on the material being processed. It is crucial to note that material behavior varies significantly under different strain rates. Consequently, selecting an appropriate material model becomes a critical factor in FEA simulations. The choice of material model directly influences the accuracy and reliability of the FEA results, ensuring that the simulated behavior aligns closely with real-world performance across varying strain rates. Therefore, careful consideration and selection of the material model are imperative to obtain meaningful and dependable insights from FEA analyses in contexts involving LSP processes [56].

In simulation software and academic research circles, the JC equation is a fundamental tool for tackling many engineering and material science problems. This equation is extensively employed to model and solve intricate scenarios, particularly those involving the dynamic behavior of materials under extreme conditions such as high strain rates and elevated temperatures. With its robust formulation, the JC equation offers a systematic approach to characterize material response, encompassing factors like strain hardening, strain rate sensitivity, and thermal softening. Researchers leverage its versatility to simulate diverse phenomena spanning from ballistic impacts to metal-forming processes, providing valuable insights into material behavior and aiding in the design of structures and systems resilient to demanding environments. As a cornerstone in computational mechanics and materials science, the JC equation continues to play a pivotal role in advancing engineering knowledge and innovation [69].

(3) $$\sigma =\left[A+B\varepsilon_{p}^{n}\right][1+∁\;In{\dot{\varepsilon }}^{*}]\left[1-\left(T^{*}\right)m\right]$$

and

(4) $$T^{*}=(T_{Test}-T_{Room})/(T_{Melt}-T_{Room})$$

where A is yield strength, B is the work hardening coefficient, C is strain rate sensitivity, n is the strain hardening coefficient, and $\varepsilon_{p}$ is plastic strain $\dot{\varepsilon }$ is strain rate and ${\dot{\varepsilon }}_{0}$ is refrence strain rate.

Therefore,

(5) $${\dot{\varepsilon }}^{*}=\dot{\varepsilon }/{\dot{\varepsilon }}_{0}$$

In technical terms, flow stress represents a critical parameter, encapsulating the combined influence of strain hardening, strain rate, and temperature within the context of a specific equation or model. Strain hardening refers to the material’s increased resistance to deformation as it is subjected to plastic strain. The strain rate signifies the speed at which deformation occurs within the material. Moreover, temperature plays a pivotal role, impacting the material’s response to deformation, with higher temperatures typically resulting in reduced flow stress due to increased atomic mobility and decreased resistance to deformation. When these factors are multiplied together within the mentioned equation or framework, the resultant flow stress value provides crucial insights into the material’s behavior under various mechanical conditions, aiding in the characterization and prediction of its mechanical properties for practical engineering applications [70]. In the framework of FEA software, the treatment of thermal effects in wave propagation is a critical consideration. The J-C model, employed in various FEA software packages, simplifies the analysis by neglecting the thermal effect of wave propagation [71]. This simplification is convenient for certain applications but may limit the accuracy of stress predictions, particularly in scenarios where thermal effects play a significant role. To address this limitation, alternative equations have been developed, such as the Zerilli-Armstrong (ZA) [72] and Khan-Huang-Liang (KHL) [73] models. These models offer more comprehensive approaches to account for thermal effects during wave propagation, allowing for more accurate determination of stress generation. By incorporating these alternative equations into FEA simulations, engineers and researchers can obtain more precise and reliable results, particularly in situations where thermal considerations are crucial. In technical terms, the computational resources required for solving the equations pertinent to the task are notably high. However, this computational burden can be alleviated significantly by optimizing the damping value employed in the process without compromising the accuracy or integrity of the Large-Scale Power Finite Element simulation. By adjusting this parameter effectively, computational efficiency can be improved, allowing for more streamlined and resource-efficient simulations while maintaining fidelity to the underlying physics and dynamics of the system under study [74].

In recent years, there has been a notable shift toward the adoption of the eigenstrain approach in the realm of stress prediction within technical fields. This methodology represents a novel and increasingly favored method for forecasting stress levels in various materials and structures. The eigenstrain approach introduces a sophisticated framework that leverages mathematical principles and computational techniques to anticipate stress distributions with greater accuracy and efficiency. By integrating concepts from linear elasticity theory and numerical analysis, this approach facilitates a comprehensive understanding of stress behaviors under different loading conditions. Moreover, its applicability spans diverse domains, ranging from mechanical engineering to materials science and beyond. As a result, the eigenstrain approach emerges as a valuable tool for engineers, researchers, and practitioners seeking precise stress predictions to inform design, analysis, and optimization processes. Its growing prominence underscores its potential to significantly advance the field of stress analysis, offering a refined and comprehensive means of addressing complex engineering challenges [75,76,77]. In technical terms, the concept of eigenstrain refers to the spatial distribution of strain within a material body that arises due to various inelastic processes. These processes can include plastic deformation, thermal expansion mismatches, or phase transformations. Eigenstrain essentially characterizes the internal strain distribution within a material caused by these factors, which can have lasting effects on the material’s mechanical properties and structural integrity. For instance, plastic deformation resulting from applied loads or temperature changes can induce eigenstrain, leading to residual stresses and potential structural weaknesses over time. Similarly, thermal expansion mismatches between different components or phases within a material can also generate eigenstrain, affecting its overall behavior and performance. Understanding and quantifying eigenstrain is crucial in various engineering applications, particularly in predicting material behavior under different operating conditions and designing resilient structures or components [78].

In the realm of computational methods, particularly within the explicit method framework, the concept of time increment stands as a pivotal factor of utmost significance. Time increment refers to the discrete intervals at which calculations are performed within the simulation. It serves as a fundamental parameter governing the temporal resolution and accuracy of the numerical model. The selection of an appropriate time increment is paramount, as it profoundly influences the reliability and precision of the computational results obtained. A judicious choice of time increment ensures that the simulation captures the dynamic behavior of the system with fidelity while simultaneously maintaining computational efficiency. Consequently, careful consideration and optimization of the time increment parameter are imperative to attain trustworthy and meaningful outcomes in engineering simulations and analyses [28]. After the choice of time increment, another critical aspect that significantly impacts the accuracy of simulation outcomes is the meshing of the model.

## 7. Influence of Meshing on Simulation Outcomes

In the researcher’s conclusion regarding FEA results, it is emphasized that achieving precise predictions requires a thorough consideration of several critical factors. Firstly, attention to constitutive modeling details is deemed essential. This involves a thorough understanding and accurate representation of the material properties and behaviors within the simulated environment. Secondly, the loading history must be carefully accounted for. This entails a comprehensive tracking and analysis of how the applied loads evolve over time, as this information significantly influences the structural response. Lastly, the importance of mesh refinement is underscored. Refining the mesh ensures that the computational domain is adequately discretized, enabling a more accurate representation of the structural geometry and enhancing the simulation results’ fidelity. Overall, these key considerations underscore the complexity and intricacy involved in obtaining reliable predictions through FEA, highlighting the necessity for thoroughness and precision in the modeling process [22,79]. The utilization of these specific mesh structures yields highly precise outcomes concerning residual stress analysis and wave-propagation assessments within technical contexts. This mesh design offers a robust framework for conducting intricate simulations and analyses, ensuring the fidelity of the results. In applications in which understanding residual stress distributions or evaluating wave-propagation characteristics is critical, this mesh configuration stands out for its ability to capture nuanced details and provide reliable data. Its effectiveness lies in its capacity to accurately represent the complex interactions inherent in these phenomena, making it an invaluable tool for technical investigations and engineering analyses [21,74,80]. In technical applications, it is paramount to recognize the significance of implementing various mesh densities across different spatial locations. This strategic approach is crucial for achieving precise outcomes while simultaneously optimizing costs and minimizing time investments. By tailoring mesh densities according to the specific requirements of each location, engineers and analysts can effectively balance computational resources with accuracy, thereby enhancing the efficiency of simulations and analyses. This practice not only ensures the fidelity of the results but also streamlines the overall computational process, enabling a more streamlined and cost-effective workflow. In essence, the judicious adoption of diverse mesh densities represents a fundamental strategy for achieving optimal outcomes in technical endeavors, striking an equilibrium between precision and resource utilization [79,81]. When addressing time-dependent problems through the utilization of solvers that iteratively update solutions until convergence is achieved, determining the appropriate increment becomes pivotal. This critical increment is intricately tied to the mesh size, which is a fundamental aspect of the numerical simulation.

It is expressed mathematically by the following equation:

(6) $$T_{inc}=0.1\times L\sqrt{\frac{\rho }{E}}$$

where, T_inc_ is critical time increment, L is mesh size, ρ is the density of the sample, and E is Young’s modulus.

This equation serves as a cornerstone in the iterative process, guiding the solver’s progression toward an accurate and reliable solution within the time-dependent domain. As such, understanding and effectively implementing this relationship is indispensable for achieving computational efficiency and accuracy in numerical simulations of dynamic phenomena [82].

Given its inherent nonlinearity and nonstatic nature, effectively modeling the LSP requires the incorporation of time-dependent or independent incremental load steps. These steps are indispensable for resolving the complexities intrinsic to the process and ensuring the accurate representation and analysis of its dynamic behavior. By leveraging such computational techniques, engineers and researchers can effectively study and optimize the effects of LSP on material properties, facilitating advancements in various technical domains reliant on this innovative surface treatment method [83]. Table 3 shows the review studies conducted using FEA for the LSP.

## 8. Progressive Perspectives: Advancement in LSP FEA

In recent years, there has been a notable surge in research aimed at integrating artificial intelligence (AI) and Machine Learning (ML) methodologies within the realm of mechanical engineering. Particularly noteworthy are the efforts directed toward leveraging these technologies to enhance various aspects of mechanical processes. One significant application has been the utilization of Artificial Neural Networks (ANNs) in predicting residual stress. ANNs, inspired by the intricate workings of the human brain, offer a powerful tool for modeling complex relationships within datasets. Researchers have successfully employed ANNs to forecast residual stress levels following LSP processes. This innovative approach circumvents the need to run multiple simulations, streamlines the prediction process, and provides valuable insights into surface state post-treatment. By harnessing the capabilities of ANNs, this methodology represents a promising advancement in the field of mechanical engineering, offering enhanced efficiency and accuracy in predicting critical outcomes [7,88].

ANNs represent a highly efficient approach for tackling complex, nonlinear problems within various domains. By leveraging interconnected layers of artificial neurons, ANNs can discern intricate patterns and relationships within data that may not be readily apparent through traditional linear methods. This inherent capacity to capture nonlinear dependencies makes ANNs particularly adept at tasks such as pattern recognition, classification, regression, and time-series prediction across diverse fields, including but not limited to machine learning, computer vision, natural language processing, and finance. By employing techniques like backpropagation and gradient descent, ANNs can iteratively adjust their parameters to optimize performance and accurately model complex data distributions. Consequently, their effectiveness in solving nonlinear problems underscores their widespread adoption and continuous advancement within the realm of computational intelligence [89,90].

In the research conducted by Sakhvadze et al. [6], the application of ANN for the prediction of Radar Cross Section (RCS) was investigated. Their study yielded promising results, with an accuracy exceeding 95% across various laser power settings. This signifies the efficacy of ANN as a predictive tool in estimating RCS, showcasing its potential for enhancing radar systems’ performance and contributing to advancements in target detection and identification technologies. The achievement of such high accuracy levels underscores the robustness and reliability of the employed ANN model in capturing the complex relationships between laser power and RCS, thereby offering valuable insights for further research and practical implementation in radar system design and operation [7].

In current research endeavors, AI has emerged as a pivotal tool for identifying residual stress within materials. Employing advanced methodologies, researchers have turned to sophisticated AI techniques, notably convolutional neural network (CNN) and Long Short-Term Memory (LSTM) models. These methodologies harness the power of AI to analyze complex datasets and discern patterns that signify residual stress within materials. By leveraging CNN’s ability to extract intricate features from raw data and LSTM’s capability to grasp temporal dependencies, researchers can achieve unprecedented accuracy and efficiency in the detection and characterization of residual stress. This fusion of AI with traditional material science methodologies marks a significant advancement in the field, promising enhanced insights and breakthroughs in understanding and managing residual stress phenomena [91,92,93].

Table 4 summarizes the various methods for performing laser shock finite element analysis, highlighting the detailed models, homogenized approaches, crystal plasticity-based simulations, and techniques for analyzing residual stresses and thermo-mechanical effects. Recent research references have been provided to support each method and their specific applications and advantages.

**Figure 6:**
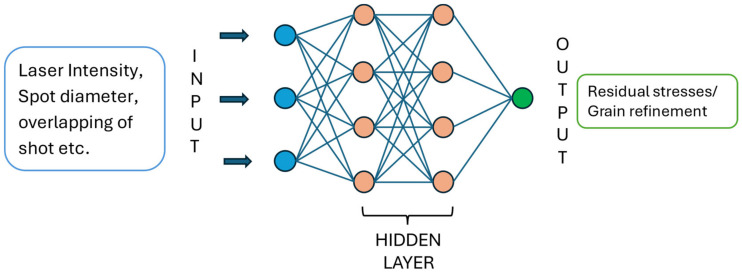
ANNs architecture with three main sections consisting of an inputs layer, hidden layers, and outputs layer, adapted [96].

Figure 6 shows the input parameters such as laser intensity, spot diameter, and overlapping of shot for ANN of LSP passes through the hidden layer and provides the output as residual stresses or grain refinement values.

## 9. Discussion

In FEA, the modeling of melting phenomena is often overlooked. Consequently, when predicting residual stresses in processes like LSP without the presence of a coating, there tends to be a significant deviation between the calculated values and those observed experimentally, particularly in the surface region. This discrepancy emphasizes the complexity of accurately simulating the intricate thermal and mechanical interactions involved in such processes, highlighting the need for further refinement of computational models to enhance predictive capabilities and bridge the gap between simulation and real-world outcomes [26]. A comparison between the experimental findings and simulation results for the aluminum alloy revealed a relative error of below 11%. The FEA model’s reliability was cross-verified using a complex surfaced model with simulations performed under specific input parameters, and the stress from the experimental LSP was analyzed using high-end techniques such as X-ray diffraction. This indicates a favorable agreement between the two methodologies, suggesting that the FE simulation outputs regarding the residual stress are reliable. Such congruence underlines the efficacy and accuracy of FE simulations in predicting and analyzing residual stresses within aluminum alloy materials. This validation enhances the confidence in utilizing FE simulations as a valuable tool for studying and optimizing the mechanical behavior of aluminum alloys in various engineering applications [97]. In addition, the results from Keller et al. [52] show that the FEA error was less than 5%. In addition to the aforementioned methods, alternative algorithms are available for constructing programs that leverage evolutionary principles. These include Genetic Algorithms [96], which simulate biological evolution to find solutions through mutation, crossover, and selection processes. Particle Swarm Optimization Algorithm [98] is another technique inspired by the collective behavior of organisms, where candidate solutions (particles) iteratively adjust their positions based on the best-performing individuals in the search space. Furthermore, the Ant Colony Optimization Algorithm mimics the foraging behavior of ants to iteratively build solutions by depositing pheromones along favorable paths, guiding the search toward optimal solutions. These diverse approaches offer varying strategies for problem solving and optimization, catering to different application domains and preferences in algorithmic design [93].

## 10. Future Direction

FEA is a way of understanding and optimizing the LSP process that we have discussed in the previous sections. Few studies have been carried out using FEA for multiphase analysis. There is an avenue for considering the thermal-mechanical effect of the LSP for the optimization process and calculating the results accurately. Recently, trends have shown data-driven processes, such as machine learning and artificial intelligence invasion, in FEA. This can be effectively utilized by combining experimental data with FEA outcomes to train algorithms that ultimately predict the LSP process more accurately. In a recent study, researchers intensively studied laser shock surface patterning [99,100,101], surface characterization for LSP’ed specimens [89], and its effect on magnesium alloys [102,103].

Intensive research has been conducted to understand the tribological and microstructural performances of raw steel specimens subjected to LSP [104]. In addition, the same performance testing was performed on specimens manufactured via additive manufacturing [105]. Researchers have studied the mechanism of stress corrosion cracking resistance in stainless steel welds subjected to LSP without coating [106,107], and the same testing was carried out on additive-manufactured specimens [108]. Few researchers have studied material grains on a microscopic level [8,48] numerically; however, there is still scope to understand the physical phenomena of dislocation theory, grain boundaries, phase transformation, etc., under dynamic conditions using FEA. There is scope for multiphase FEA analysis on a microscopic level to enable a comprehensive understanding of the LSP phenomenon and its effects on the material. Mostly, FEA is performed independently by combining numerical calculations and input parameters to understand phenomena. Therefore, there is a new avenue for combining experimental outputs with FEA inputs to reduce FEA efforts. This combination can lead to more accurate and reliable results for LSP effects. In previous research, FEA analysis was performed without considering the confinement layer over the specimen and neglecting chemical reactions taking place on the surface. Adaptive time-stepping techniques and subcycling can be implemented to stabilize the results.

## 11. Summary

The present work in this review paper delves into the complexities of the LSP process, emphasizing its simulation through FEA. Compared with conventional surface modification techniques, LSP is the best alternative option available to generate a deeper compressive residual stress field and the formation of a gradient nanostructure in the surface layer to prevent crack initiation in metallic components. It carefully examines various FEA approaches and their outcomes and highlights the important considerations necessary for accurate modeling and simulation of the LSP process. It focuses on various material model constitutive models, such as Johnson–Cook, Zerilli-Armstrong, etc., for reliable simulations. Crucial parameters, including the laser power and overlapping of shots, are discussed to analyze their effect on the induced residual stress and surface properties. Optimizing these parameters using a simulation technique can lead to the required surface properties. LSP processes have evolved over the last decade, and advancements in the inclusion of AI and Machine Learning have also been reviewed. This inclusion should focus on integrating experimental outputs with FEA inputs to reduce computational effort and enhance accuracy. Although this review presents the simulation techniques and recent advancements in this field, addressing the actual parameter selection remains a challenge.

## Figures and Tables

**Table 1:** Effects of the laser parameters on the material properties.

Laser Parameter	Material	Effect on Material Properties	Reference
Laser Energy (Joule)	Inconel 718 Super-alloy	- Increased high-temperature fatigue resistance - Enhanced creep resistance	[51]
Pulse Duration (ns)	AA2198-T3 AA2198-T8	- Improved resistance to crack initiation - Increased surface hardness	[52]
Spot Size (mm)	AA2198-T8 AA2198-T8	- Enhanced fatigue performance - Improved stress corrosion resistance	[52]
Number of Pulses	IN718 Alloy (Inconel 718)	- Increased resistance to fretting fatigue - Enhanced creep resistance	[51]
Laser Wavelength (nm)	7075 Aluminum Alloy	- Improved resistance to crack initiation - Enhanced material toughness	[46]
Laser Intensity (GW/cm^2^)	AA2198-T3 AA2198-T8	- Enhanced tensile strength - Reduced surface roughness	[52]

**Table 2:** Simulation and experimental method comparison of different aspects.

Aspect	Simulation Results	Experimental Results	Comparison	Reference
Residual Stress Profile	Predicts compressive residual stresses to a depth of 1 mm	Measured residual stresses show similar depth but vary in magnitude	Simulation predicts trends accurately; slight magnitude differences may suggest refinement in material modeling needed.	[52]
Plastic Deformation Depth	Predicted plastic zone depth: 1 mm	Measured plastic zone depth: 2 mm	Good agreement; validates the accuracy of the simulation model.	[52]
Temperature Rise	In most work, LSP is assumed to be a purely mechanical process. Therefore, the influence of the temperature is assumed to be negligible.	Experimental temperature increases up to 48 °C	Close match; confirms thermal aspects of simulation.	[52]
Crack Formation/Propagation	Predicts reduced crack propagation rate due to compressive stresses	Experiment confirms reduced crack growth but at a slower rate than predicted [10].	Simulation trends confirmed; rate differences highlight possible factors like microstructural effects not fully captured.	[10,52]
Process Efficiency	Simulated process efficiency under optimal conditions	Experiment shows efficiency slightly lower than predicted	Efficiency loss in real conditions may be due to unmodeled factors like energy losses.	[52]
Effect of Transparent Overlays & Absorbent Coatings	FE model shows enhanced shock response (SRS amplitude) with minimal impact on SRS slope.	Experimental tests confirm increased SRS amplitude and negligible effect on SRS slope.	Both methods confirm that coatings improve shock response amplitude, validating the FE model’s accuracy.	[50]
Capacity to Simulate Transient Response	FE model accurately simulates the transient response of the aluminum alloy plate under laser shock.	Experimental data supports the model, showing consistent transient response characteristics.	Strong correlation indicates the FE model’s reliability in predicting transient dynamics.	[50]
Shock Response Spectrum (SRS) Amplitude	FE model predicts SRS amplitude reaching hundreds of g, with a high and broadband frequency range.	Experimental results confirm high SRS amplitudes and similar frequency characteristics.	The FE model effectively captures the high SRS amplitude and frequency behavior observed experimentally.	[50]
Relationship Between Laser Parameters and Response	Simulated response increases linearly with peak pressure and shows a quadratic relationship with laser power density.	Experimental data corroborates the linear increase with peak pressure and quadratic correlation with power density.	Both approaches demonstrate the same trends, validating the predictive capability of the FE model.	[50]
Effect of Pulse Duration and Spot Size on Response	FE model shows enhanced response with longer pulse durations and larger spot sizes, with complex trends between 100 Hz and 10,000 Hz.	Experiments confirm enhancement at extreme frequencies and complex, non-monotonous behavior in the mid-frequency range.	The FE model accurately reflects the experimental trends, although complexities in mid-frequency behavior warrant further study.	[50]
Effect on SRS Slope	FE model indicates that laser pulse duration significantly affects SRS slope, while spot size has minimal impact.	Experimental results support the significant effect of pulse duration on SRS slope, with little influence from spot size.	Consistent findings across both methods affirm the FE model’s precision in capturing the effects on SRS slope.	[50]

**Table 3:** Review of studies conducted using the FEA of LSP.

Material	Software’s Used	Variable Parameters Considered	Reference
Ti-6Al-4V alloy	Abaqus	The effect of the spot size and shape, the pulse energy, the number of peen layers, overlapping of spots, and temporal variation of the mechanical pressure induced by plasma is considered and analyzed	[84]
Inconel 718 super-alloy	Abacus Explicit and Implicit PSO technique with Python coding	Laser power, Laser beam shape, Scan Pitch, Scan Pattern	[51]
TC4 alloy	Abaqus Explicit and Standard	Shock angle, laser polarization state	[85]
Ti6Al4V titanium alloy	Abaqus Standard	Laser power, Impact times, Pulse duration, Spot size	[86]
AA2198-T3 AA2198-T8	Abaqus Explicit and Standard	Laser power, square laser beam dimension, Temperature, Thickness	[52]
7075 aluminum alloy	Abaqus/Explicit	Scanning patterns, Overlapping rate, Spot shape (Square and Circle).	[46]
STS304	ANSYS Autodyn	Number of shots, Damping value	[74]
Pure Al	Abaqus/CAE FEM	Material thickness, Spot diameter, Laser power	[87]
IN718 alloy	Abaqus and MSC.Patran	Laser power, Temperature	[12]

**Table 4:** Recent methods to perform FEA of LSP.

Method	Description	References
Detailed FE Models	Models the geometry and material properties in detail, allowing for accurate simulation of shock wave propagation.	[94]
Homogenized FE Models	Uses averaged material properties to simplify the model and reduce computational time, though with less accuracy.	[94]
Crystal Plasticity-Based FE Models	Incorporates crystal plasticity theories to simulate deformation mechanisms at the grain level.	[95]
Explicit FE Analysis with LS-Dyna	Employs explicit dynamics software like LS-Dyna to simulate high-strain-rate behaviors and shock responses.	[94]
Residual Stress Analysis	Focuses on analyzing and mitigating residual stresses induced by laser peening in thin structures.	[96]
Progressive Damage Models	Applies damage mechanics to predict initiation and propagation of cracks and other failures in the material.	[94]
Thermo-Mechanical Coupled Analysis	Considers both thermal and mechanical effects simultaneously to capture the complex interactions during shock.	[96]
High-Fidelity 3D Woven Composite Models	Utilizes high-fidelity models to represent the complex architecture of woven composites for more accurate results.	[94]
Simulation of Plasma Expansion Effects	Models the expansion of plasma created by laser interaction to understand its impact on material deformation.	[94]

## Data Availability

The original contributions presented in the study are included in the article, further inquiries can be directed to the corresponding author.
